# Prognostic Value of the De Ritis Ratio in Predicting Survival After Bladder Recurrence Following Nephroureterectomy for Upper Urinary Tract Tumors

**DOI:** 10.3390/diagnostics15151840

**Published:** 2025-07-22

**Authors:** Enis Mert Yorulmaz, Kursad Donmez, Serkan Ozcan, Osman Kose, Sacit Nuri Gorgel, Enes Candemir, Yigit Akin

**Affiliations:** 1 Department of Urology, Izmir Katip Celebi University, Izmir 35620, Turkey; drserkanozcan@hotmail.com (S.O.); oskose@gmail.com (O.K.); sngorgel@hotmail.com (S.N.G.); candemirenes@gmail.com (E.C.); yigitakin@yahoo.com (Y.A.); 2 Department of Urology, Ataturk Research and Training Hospital, Izmir 35360, Turkey; kursad1@gmail.com

**Keywords:** De Ritis ratio, intravesical recurrence, nephroureterectomy, prognostic biomarker, upper tract urothelial carcinoma

## Abstract

**Background/Objectives**: Upper tract urothelial carcinoma (UTUC) is often complicated by intravesical recurrence and cancer progression following radical nephroureterectomy (RNU). Identifying reliable prognostic biomarkers remains crucial for optimizing postoperative surveillance. The goal of this study was to assess the prognostic value of the De Ritis ratio (AST/ALT) in predicting bladder recurrence and oncologic outcomes in patients with clinically localized UTUC undergoing RNU. **Methods**: This retrospective study analyzed 87 patients treated with RNU between 2018 and 2025. Preoperative De Ritis ratios were calculated, and an optimal cut-off value of 1.682 was determined using ROC analysis. Recurrence-free survival (RFS), cancer-specific survival (CSS), and overall survival (OS) were analyzed using the Kaplan–Meier and Cox regression methods. Logistic regression was used to identify independent predictors of bladder recurrence. **Results**: A high De Ritis ratio was significantly associated with increased bladder recurrence and worse RFS and CSS, but not OS. Multivariate analysis confirmed that an elevated De Ritis ratio, current smoking, positive surgical margins, and synchronous bladder cancer were the independent predictors of bladder recurrence. The De Ritis ratio demonstrated strong discriminatory performance (AUC: 0.807), with good sensitivity and specificity for predicting recurrence. **Conclusions**: The De Ritis ratio is a simple, cost-effective preoperative biomarker that may aid in identifying UTUC patients at higher risk for intravesical recurrence and cancer-specific mortality. Incorporating this ratio into clinical decision-making could enhance risk stratification and guide tailored follow-up strategies.

## 1. Introduction

Upper urinary tract urothelial carcinoma (UTUC) is a relatively rare malignancy, accounting for only 5% to 10% of all urothelial carcinomas [1,2]. However, recent studies have indicated a rising incidence of UTUC [3]. Radical nephroureterectomy (RNU), including bladder cuff excision, is currently regarded as the standard treatment for localized UTUC [4]. Nevertheless, postoperative systemic disease recurrence remains a significant concern. Up to 30% of patients, particularly those with advanced-stage disease, experience tumor recurrence and cancer-related mortality despite surgical intervention [5,6]. Despite surgical precautions, intravesical recurrence after RNU remains a common occurrence, affecting approximately 22% to 47% of patients [7,8].

Consequently, various preoperative prognostic models have been developed to optimize individualized treatment approaches and surveillance strategies for UTUC patients [1,9]. Aspartate aminotransferase (AST) and alanine aminotransferase (ALT) are enzymes released into the bloodstream from hepatocytes as biomarkers of hepatocellular injury [10]. These enzymes are widely included in routine biochemical panels for liver function assessment [11]. The De Ritis ratio, initially described by De Ritis, represents the ratio of AST to ALT and has been utilized as a prognostic biomarker in differentiating liver diseases and predicting postoperative survival outcomes in various malignancies [12,13,14,15]. In recent years, emerging evidence has suggested that altered aminotransferase levels, including the De Ritis ratio, may serve as valuable prognostic indicators in specific cancer types. While some studies have proposed a potential prognostic role of the De Ritis ratio in UTUC, the rarity of the disease and the limited sample sizes in existing studies have hindered the establishment of a definitive correlation [16,17,18].

UTUC and bladder cancer (BCa) share similar biological, histological, and pathological characteristics. Intravesical recurrence is a frequent event and remains one of the primary concerns during the follow-up of patients who have undergone RNU [19].

In this study, we conducted a retrospective analysis of the clinicopathological data of patients with clinically localized UTUC who underwent RNU, aiming to evaluate the prognostic significance of aminotransferase levels in survival outcomes. Additionally, we sought to investigate the factors influencing bladder recurrence in these patients and to explore the potential role of the De Ritis ratio in this context.

## 2. Materials and Methods

### 2.1. Study Design and Population

This retrospective cohort study analyzed 87 patients who were diagnosed with clinically localized UTUC and underwent RNU between January 2018 and December 2025 at Izmir Katip Çelebi University.

Eligible patients had a histologically confirmed diagnosis of UTUC with clinical T stage of T2 or lower, no evidence of lymph node involvement or distant metastasis on preoperative imaging, and had undergone RNU with curative intent. Patients with histologically confirmed conventional urothelial carcinoma were included. Cases with variant histology were excluded from the final analysis. Only patients with available preoperative serum AST and ALT measurements suitable for De Ritis ratio calculation were included in the study cohort.

Patients were excluded if they had a prior or concurrent history of BCa; previous or synchronous contralateral UTUC; underlying chronic liver diseases such as viral hepatitis, cirrhosis, or non-alcoholic steatohepatitis; or if they had been using hepatotoxic medications within three months prior to surgery. Patients exhibiting significant abnormalities in liver function tests unrelated to UTUC, experiencing perioperative mortality within 30 days after surgery, or lacking sufficient clinicopathological or follow-up data were also excluded.

Demographic characteristics, perioperative details, pathological findings, and survival outcomes were retrospectively collected through a comprehensive review of institutional electronic medical records.

All the patients provided informed consent prior to surgery. The study protocol was reviewed and approved by the Institutional Ethics Committee (2025-SAEK-0410).

The primary objective of this study was to assess the prognostic significance of the De Ritis ratio (serum AST to ALT ratio) in predicting recurrence-free survival (RFS), cancer-specific survival (CSS), and overall survival (OS) following RNU for clinically localized UTUC.

### 2.2. Surgical Procedures

All the patients underwent RNU performed via either an open or a laparoscopic approach depending on surgeon preference and tumor characteristics. In all the cases, the distal ureter and the bladder cuff were excised en bloc to ensure oncological radicality. For laparoscopic RNU, the procedure was initiated with standard port placement under general anesthesia. Following the mobilization of the kidney and ureter, the distal ureter and bladder cuff were managed using either an endoscopic stapling device or open excision after repositioning the patient according to intraoperative findings. Open RNU was performed through a standard flank or transperitoneal incision with the meticulous dissection of the ureter down to its intramural portion, followed by the open excision of the bladder cuff.

Regardless of the surgical approach, the excision of the bladder cuff was performed either by endoscopic transurethral resection or by the open surgical technique, with the complete removal of the intramural ureter and adjacent bladder wall. Special attention was given to avoid tumor spillage during specimen retrieval.

The decision to perform a lymphadenectomy was made based on the surgeon’s intraoperative judgment, considering the tumor location, imaging findings, and intraoperative evaluation. Dedicated genitourinary pathologists evaluated all the surgical specimens using the 2017 TNM classification and WHO grading system for urothelial carcinoma.

### 2.3. Follow-Up Protocol

Postoperative surveillance was conducted according to the contemporary European Association of Urology (EAU) Guidelines applicable during the study period. The patients were evaluated every three months during the first postoperative year, followed by every six months during the second year, and annually thereafter. Each follow-up visit included a detailed physical examination, serum chemistry panel, urine cytology, and cystoscopic evaluation of the bladder. Annual diagnostic imaging of the contralateral upper urinary tract was performed using cross-sectional modalities such as computed tomography urography, ultrasonography, or intravenous pyelography to monitor for metachronous UTUC. CT urography was preferred for upper tract imaging when clinically feasible; however, intravenous pyelography or ultrasonography was used in patients with contraindications to contrast-enhanced imaging, including impaired renal function or contrast allergy. Chest radiography was routinely conducted at each follow-up, and chest computed tomography or bone scans were performed at the attending physician’s discretion when clinically indicated.

Recurrence was defined as any evidence of local tumor relapse in the retroperitoneal region or renal fossa or as distant metastasis detected radiographically or pathologically. Intravesical recurrence was considered as bladder recurrence and recorded separately.

All causes of death were confirmed through a thorough review of hospital records, physician documentation, and, when necessary, death certificates. The patients with unknown survival status were verified via hospital communication systems or direct telephone contact.

### 2.4. Definition of Outcomes

RFS was defined as the time from the date of RNU to the occurrence of any recurrence, including local relapse in the retroperitoneal region or renal fossa, distant metastasis, or intravesical recurrence within the bladder. The patients who remained free of recurrence at the time of the last follow-up were censored. CSS was defined as the time from RNU to death, specifically attributable to UTUC. Deaths from causes unrelated to UTUC were censored at the date of death. OS was defined as the time from RNU to death from any cause. The patients who were alive at the last follow-up were censored at that time.

### 2.5. Statistical Analysis

Continuous variables were presented as mean ± standard deviation (SD) or median with interquartile range (IQR) based on the assessment of normality using the Shapiro–Wilk test. Categorical variables were expressed as frequencies and percentages. Comparisons between groups were performed using the chi-square test or Fisher’s exact test for categorical variables, and Student’s *t*-test or Mann–Whitney U test for continuous variables, as appropriate. The optimal De Ritis ratio cut-off for group stratification was determined using a ROC curve analysis, yielding a threshold of 1.682 for predicting bladder recurrence. RFS, CSS, and OS were estimated using the Kaplan–Meier method, and differences between survival curves were assessed with the log-rank test. Univariable and multivariable Cox proportional hazards regression analyses were performed to identify independent prognostic factors for RFS, CSS, and OS. Variables with a *p*-value < 0.10 in the univariable analyses were included in the multivariable models. Hazard ratios (HRs) and 95% confidence intervals (CIs) were calculated. The proportional hazards assumption was verified using Schoenfeld residuals and graphical inspection. Model discrimination was assessed using the concordance index (C-index).

All the statistical analyses were performed using the Jamovi software (Version 2.6.17.0; Sydney, Australia). A two-tailed *p*-value < 0.05 was considered statistically significant.

## 3. Results

### 3.1. Patient Characteristics

The study included 87 patients who underwent RNU for clinically localized UTUC. The patients were stratified according to the presence or absence of subsequent bladder recurrence. Bladder recurrence occurred in 26 (29.9%) patients. Female sex was significantly associated with a lower risk of recurrence, as all recurrence cases were observed in male patients (*p* = 0.017). Smoking status also showed a strong association, with current smokers having a significantly higher recurrence rate than non-smokers (43.2% vs. 9.3%, *p* < 0.001). Obesity was more common among patients with recurrence (57.9% vs. 42.1%), while normal BMI was associated with a significantly lower recurrence rate (7.0%, *p* < 0.001). Although not statistically significant, diabetes mellitus showed a trend toward higher recurrence (37.5% vs. 20.0%, *p* = 0.074). Other comorbidities, such as hypertension, coronary artery disease, and alcohol use, did not demonstrate a significant difference between the groups. Baseline demographic features are presented in Table 1.

The comparison of preoperative laboratory values according to bladder recurrence status is summarized in Table 2. There were no statistically significant differences between the groups regarding age, hemoglobin, white blood cell count, platelet count, and creatinine (*p* > 0.05). However, the patients with recurrence had a significantly higher body mass index (BMI) compared to those without recurrence (29.9 ± 3.7 vs. 24.1 ± 4.2; *p* < 0.001). De Ritis ratio was significantly elevated in the patients with bladder recurrence (2.09 ± 0.84 vs. 1.27 ± 0.63; *p* < 0.001).

Lymphovascular invasion (LVI) was significantly more common in the patients with bladder recurrence than those without (44.1% vs. 15.1%, *p* = 0.003). Positive surgical margins were also more common in the recurrence group (60.0% vs. 19.4%, *p* = 0.001).

Synchronous BCa was present in 48.6% of the patients with recurrence compared to only 11.5% of those without (*p* < 0.001). Furthermore, the patients with multiple synchronous bladder tumors had a significantly higher recurrence rate compared to those with a single tumor (*p* = 0.002). However, no difference was observed in terms of recurrence with Synchronicity BCa Stage (*p* = 0.325). Recurrence was less in the patients receiving intracavitary medication (ICM), including BCG instillation, but the number of cases was limited (*p* = 0.020).

No statistically significant relationship was observed with the UTUC stage regarding recurrence (*p* = 0.335). Recurrence was observed more frequently in high-grade tumors but was not statistically significant (low-grade 25.0%, high-grade 28.1%, *p* = 0.784, respectively). Similarly, no significant difference was found in lymph node invasion status (*p* = 0.820).

The surgical technique (open vs. laparoscopic) and bladder cuff excision method (open vs. endoscopic) also did not show any significant difference between the groups (*p* = 0.267, *p* = 0.731, respectively). Table 3 details the perioperative and pathological features of the study cohort, stratified by bladder recurrence status.

### 3.2. Predictors of Bladder Recurrence

A multivariate logistic regression analysis was performed to identify the independent predictors of bladder recurrence among the variables found to be significant in univariate comparisons. The results are presented in Table 4. In multivariate logistic regression, current smoking, elevated De Ritis ratio, positive surgical margins, and the presence of synchronous BCa remained as the independent predictors of bladder recurrence (all *p* < 0.05). The model demonstrated good fit (Deviance: 57.6; AIC: 67.6) and explained a considerable proportion of variance (McFadden R^2^ = 0.427; Tjur R^2^ = 0.469).

### 3.3. Survival Analyses

**Figure 1:**
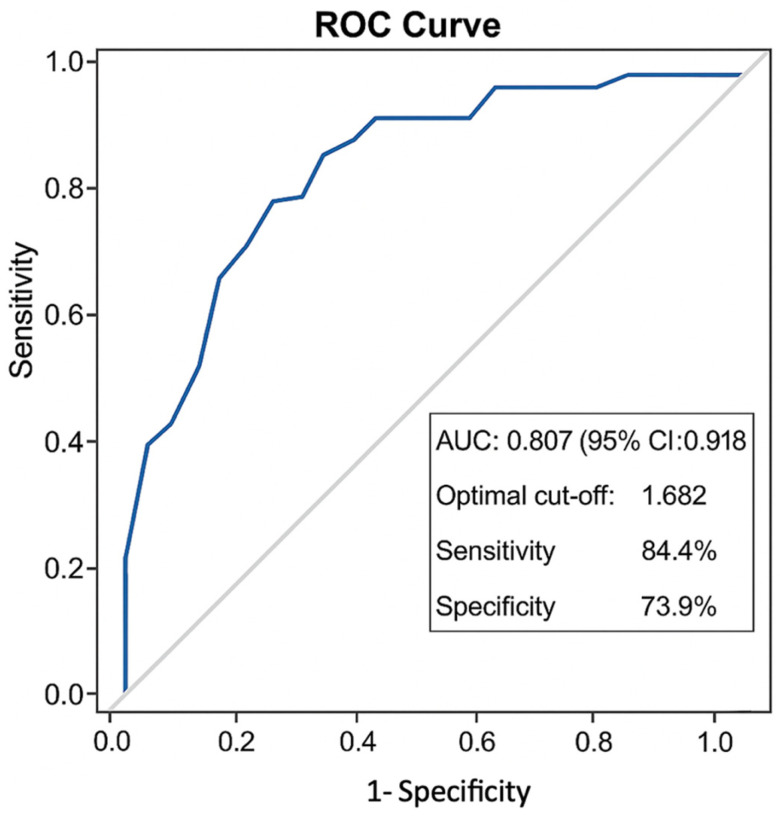
Receiver operating characteristic (ROC) curve of the De Ritis ratio for predicting bladder recurrence. The area under the curve (AUC) was 0.807 (95% CI: 0.696–0.918). The optimal cut-off value was determined as 1.682, providing 84.4% sensitivity and 73.9% specificity.

The De Ritis ratio’s prognostic performance for predicting oncologic outcomes was evaluated using survival analysis methods. The receiver operating characteristic (ROC) curve demonstrated a good discriminatory ability for predicting bladder recurrence, with an area under the curve (AUC) of 0.807 (95% CI: 0.696–0.918). The optimal cut-off value was 1.682, yielding a sensitivity of 84.4% and a specificity of 73.9% (Figure 1).

**Figure 2:**
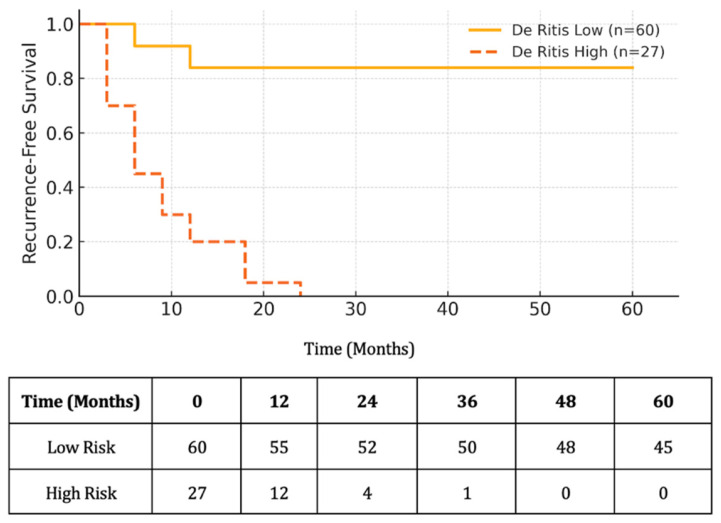
Kaplan–Meier curve for RFS stratified by De Ritis ratio (cut-off: 1.682). The patients in the high De Ritis group (n = 27) demonstrated significantly lower RFS compared to the low De Ritis group (n = 60) (log-rank *p* < 0.001). Censored cases are indicated by vertical ticks. The number at risk is shown at selected time points below the x-axis.

Based on this threshold, the patients were subsequently categorized into low and high De Ritis groups. The Kaplan–Meier survival curves showed that the patients with a high De Ritis ratio had significantly shorter RFS compared to those with a low ratio (log-rank *p* < 0.001; Figure 2). At 5 years, the RFS rate was 84.0% in the low group versus 0.0% in the high group.

**Figure 3:**
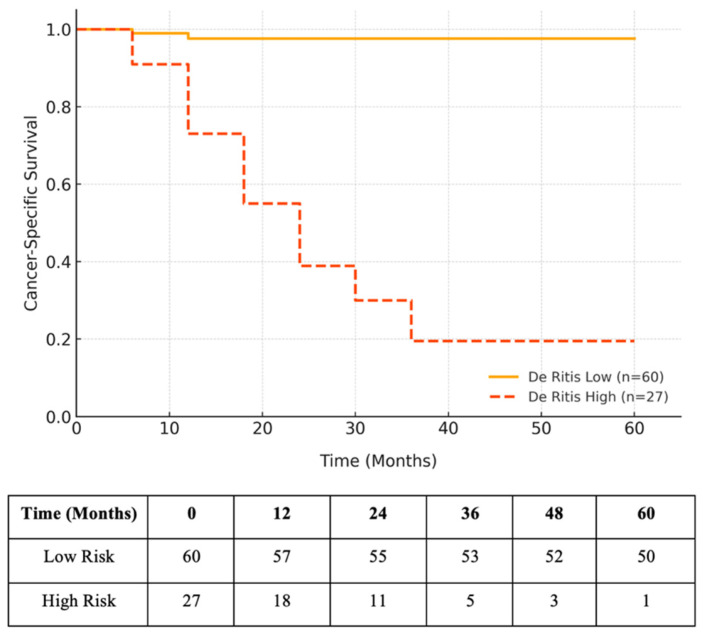
Kaplan–Meier curve illustrating CSS according to De Ritis ratio stratification. The patients in the high De Ritis group (n = 27) demonstrated significantly lower CSS compared to those in the low De Ritis group (n = 60), with 5-year CSS rates of 19.5% and 97.6%, respectively (log-rank *p* < 0.001).

CSS was significantly worse in the high De Ritis group (5-year CSS: 19.5% vs. 97.6%; log-rank *p* < 0.001; Figure 3.

**Figure 4:**
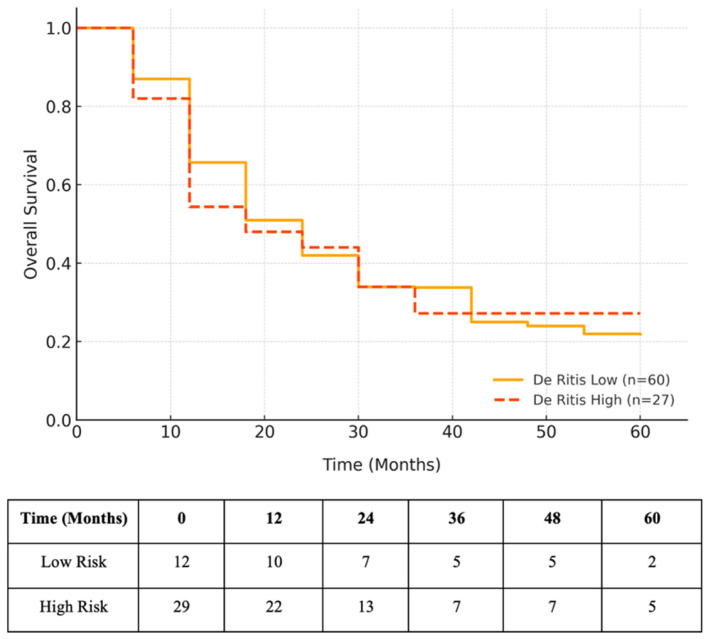
Kaplan–Meier curve depicting OS stratified by De Ritis ratio. The patients in the high De Ritis group (n = 27) showed slightly lower OS compared to those in the low group (n = 60), although the difference was not statistically significant (log-rank *p* = 0.511; HR: 1.28, 95% CI: 0.62–2.64).

OS did not differ significantly between the groups (5-year OS: 27.2% vs. 21.9%; log-rank *p* = 0.511; Figure 4.

Table 5 presents the survival estimates and hazard ratios derived from univariable Cox regression analysis. The De Ritis ratio was identified as an independent prognostic marker for RFS and CSS but not for OS.

## 4. Discussion

The present study underscores the relevance of the De Ritis as a prognostic biomarker in urothelial malignancies [15,20]. This readily available index, initially described in hepatology, has emerged as a significant predictor of outcomes in urologic oncology. Multiple studies and meta-analyses have demonstrated that an elevated AST/ALT ratio portends adverse oncologic outcomes across various urologic cancers; our findings add to this body of evidence by specifically evaluating patients with UTUC who develop bladder tumor recurrences after RNU [21]. Consistent with the literature, we found that a high preoperative De Ritis ratio (cut-off 1.682) was associated with more aggressive disease behavior, manifested as significantly worse RFS and CSS in our cohort. In contrast, OS did not differ significantly by AST/ALT status, which may reflect the influence of competing non-cancer mortality or the relatively limited follow-up period. Notably, on multivariate analysis, the De Ritis ratio remained an independent predictor of bladder recurrence risk alongside other risk factors (current smoking status, positive surgical margins, and synchronous bladder tumors), highlighting its potential value in risk stratification beyond standard clinicopathologic features.

Although UTUC diagnosis has become more accurate with the widespread use of cross-sectional imaging and ureteroscopic biopsy, serum biomarkers remain relevant in clinical decision-making. Particularly in settings where advanced imaging is not readily available or in patients with inconclusive radiological findings, the De Ritis ratio may serve as a cost-effective adjunct to conventional risk stratification tools.

Our results align with and extend prior findings in UTUC. Lee et al. (2017) were among the first to report that a high AST/ALT ratio is linked to worse survival in UTUC patients after surgery [22]. Similarly, Cho and colleagues identified an optimal AST/ALT cut-off of ~1.6 in UTUC, above which patients had significantly shorter CSS and OS, with the elevated ratio independently predicting a ~2.5-fold higher risk of cancer-specific mortality [23].

Mori et al. validated the prognostic significance of the De Ritis ratio in a large, multi-institutional UTUC cohort (n = 2492) and showed that an elevated ratio (>1.35) was associated with more aggressive pathological features such as high-grade tumors, LVI, and nodal metastasis [16]. Although their multivariable analyses did not retain the De Ritis ratio as an independent predictor for survival endpoints (RFS, CSS, and OS), their preoperative models confirmed its predictive value for adverse pathological features. Our study builds upon this by demonstrating that the De Ritis ratio remained a significant independent predictor of bladder recurrence risk even after adjusting for standard prognostic factors. This discrepancy might reflect differences in study populations, endpoints, or cut-off values (1.682 in our study vs. 1.35 in theirs) and highlights the need for context-specific interpretation.

Our finding is supported by Su et al. (2020), who, in a comprehensive meta-analysis, confirmed that a higher pretreatment AST/ALT ratio is significantly associated not only with inferior OS and CSS but also with higher likelihood of disease recurrence, including intravesical (bladder) recurrence, in urothelial carcinoma [21]. Taken together, the evidence strongly indicates that an elevated De Ritis ratio serves as a marker of biologically aggressive UTUC, predisposing patients to early recurrence and cancer-specific mortality.

Beyond UTUC, the adverse prognostic significance of the De Ritis ratio has been observed in other urothelial cancer contexts, which bolsters the generalizability of our findings. In muscle-invasive BCa, for example, Ghahari et al. (2022) reported that patients with a high AST/ALT ratio (≥1.3) had markedly worse survival outcomes following radical cystectomy [20]. In their series, the high-ratio group’s mean OS was only ~18 months compared to ~41 months in patients with a normal ratio, and the De Ritis ratio emerged as the sole independent predictor of OS on multivariate analysis [20]. Such results mirror our observation that AST/ALT can identify urothelial carcinoma patients at elevated risk of cancer-related death. A similar trend has been noted by other investigators studying BCa, prostate cancer, and renal cell carcinoma, making it increasingly clear that the De Ritis ratio reflects an underlying aspect of tumor biology that transcends individual tumor sites [15,24,25]. Our contribution specifically emphasizes its role in predicting bladder tumor relapse after UTUC, a clinically significant event that impacts patient surveillance and management.

The biological mechanisms underlying the link between AST/ALT elevation and aggressive oncologic behavior are an area of active investigation. AST and ALT are key enzymes in amino acid and carbohydrate metabolism, and emerging evidence suggests that their imbalance may promote a metabolic milieu favorable to cancer progression [26]. Rapidly proliferating tumors often exhibit the “Warburg effect,” relying on aerobic glycolysis for energy production and biosynthesis [27]. In this context, an increased AST/ALT ratio could indicate a shift toward anaerobic glycolysis and glutamine-driven TCA cycle activity in tumor cells [28]. AST and ALT play an integral role in the malate–aspartate shuttle, which transfers NADH equivalents into mitochondria to fuel glycolysis and anabolic processes [29]. Thus, a high De Ritis ratio might directly reflect these metabolic adaptations—for instance, higher AST activity (relative to ALT) could signal enhanced aspartate shuttling to support the nucleotide and amino acid synthesis required for tumor growth [21]. This shift likely marks an environment conducive to tumor proliferation, invasion, and resistance to apoptosis. Therefore, a high De Ritis ratio may be a surrogate indicator of aggressive tumor biology rather than merely reflecting hepatic dysfunction. Although our study excluded individuals with known chronic liver diseases to minimize confounding, it is impossible to completely correct for subtle changes in liver enzyme dynamics or other systemic factors. On the other hand, although patients with known liver disease or hepatotoxic drug use were excluded, subtle variations in AST and ALT levels may still arise from non-cancer-related physiological or metabolic factors, potentially influencing the De Ritis ratio. Further translational research is needed to clarify whether the AST/ALT ratio merely correlates with aggressive disease or whether it plays a more direct role in tumor progression (e.g., by facilitating gluconeogenesis or glutamine metabolism utilized by tumors). Understanding this biological link may open avenues for new interventions or risk-stratification tools, and our results may provide a pathway for these hypotheses.

From a clinical perspective, these findings have practical implications for patient management. Preoperatively identifying UTUC patients at increased risk of bladder recurrence and cancer-specific death can help tailor surveillance and adjunctive treatment strategies. For instance, the current guidelines already recommend more intensive follow-up after RNU in high-risk UTUC—with frequent cystoscopic evaluations in the first years—to promptly detect and treat bladder recurrences [30,31].

Although platinum-based chemotherapy is recommended for patients with ≥pT2 UTUC, our study cohort consisted of clinically localized cases (≤cT2) in which systemic therapy is not uniformly indicated. In this context, the De Ritis ratio may help stratify recurrence risk and guide surveillance strategies even among patients not routinely receiving chemotherapy. This marker could thus provide additional prognostic granularity in patients who are managed surgically without neoadjuvant or adjuvant therapy.

Patients with an elevated De Ritis ratio may warrant more vigilant surveillance, given their propensity for early recurrence. In our cohort, a high AST/ALT ratio was as predictive of intravesical recurrence as traditional risk factors like positive margin status or synchronous bladder tumor, suggesting it could be used as part of a risk-stratified follow-up schedule. Furthermore, recognizing a patient as “high-risk” based on AST/ALT could influence decisions on adjuvant therapy. While the standard of care for UTUC is evolving (e.g., perioperative chemotherapy for locally advanced disease), the De Ritis ratio might be factored into multi-parameter nomograms to identify patients who could benefit from clinical trial enrollment or early systemic therapy. Mori et al. have advocated incorporating the AST/ALT ratio into prognostic models to guide treatment selection, and our data support this approach [16]. For example, a patient with high AST/ALT might be counseled that they have a higher chance of tumor recurrence, prompting consideration of intravesical chemotherapy instillation after surgery or closer imaging surveillance for systemic relapse. In summary, the De Ritis ratio adds a piece of information that could refine how we risk-stratify UTUC patients and personalize their postoperative management plan.

Finally, several limitations of this study must be acknowledged. First, our analysis is retrospective and derives from a single-center experience, which introduces inherent selection biases and limits the generalizability of the conclusions. Our institution’s patient population and management protocols may not reflect those of other centers. Despite its promising prognostic associations, the De Ritis ratio has not yet been validated in prospective, multicenter studies. Therefore, its current utility in routine clinical practice remains limited. Further external validation is warranted before this marker can be incorporated into standardized risk assessment protocols. Second, the sample size is modest, reducing statistical power to detect slight differences—this may partly explain why the De Ritis ratio did not correlate with OS in our series, even though a trend toward worse survival was observed. While the retrospective nature and relatively small cohort size are acknowledged limitations, our strict exclusion criteria and complete clinical data collection from a high-volume tertiary center lend strength to the internal validity of the findings. Third, our study lacks external validation; we have not tested the AST/ALT cut-off of 1.682 in an independent cohort. Therefore, while this threshold showed clear prognostic discrimination in our data, it should be validated prospectively or in multi-center datasets before broad clinical application. Furthermore, while our findings suggest potential utility of the De Ritis ratio in early-stage disease (e.g., cT1/pT1), this hypothesis remains speculative and warrants confirmation in externally validated datasets. In addition, molecular profiling was not available due to the retrospective design and lack of standardized tissue processing, which limited a deeper understanding of underlying tumor biology.

Beyond its prognostic value, the De Ritis ratio may also reflect a patient’s physiological reserve and potential surgical risk. Although perioperative complications were not assessed in our study, this potential warrants investigation in future prospective trials.

Additionally, as with any retrospective study, we could not account for all the potential confounders (e.g., unmeasured comorbidities affecting liver enzymes), and causation could not be definitively established. These limitations temper our findings. Future research should ideally involve a larger, multi-institutional cohort with a prospective design to confirm the independent prognostic value of the De Ritis ratio in UTUC and to determine how best to integrate this biomarker into clinical decision-making. Despite these caveats, our study contributes to a growing evidence base suggesting that preoperative AST/ALT is an accessible biomarker with meaningful prognostic information in UTUC patients, particularly regarding the risk of bladder recurrence and CSS.

## 5. Conclusions

We demonstrated that the preoperative De Ritis ratio is a significant and independent predictor of intravesical recurrence and CSS in patients with clinically localized UTUC undergoing RNU. Our findings highlight the prognostic utility of this readily available and cost-effective biomarker, which may reflect underlying tumor aggressiveness and systemic metabolic alterations. Notably, the De Ritis ratio maintained its predictive strength alongside established risk factors such as smoking status, surgical margin positivity, and synchronous BCa. Incorporating the De Ritis ratio into clinical risk models could facilitate more individualized follow-up strategies and inform decisions regarding adjuvant therapy or early intervention. Future prospective multicenter studies are warranted to validate these findings and to establish the De Ritis ratio as a standard component of preoperative assessment in UTUC patients.

## Figures and Tables

**Table 1:** Baseline demographic characteristics according to bladder recurrence status.

	Bladder Recurrence		
	No (n, %)	Yes (n, %)	*p*-Value
Gender			
Male	50 (68.5%)	23 (31.5)	0.017 *
Female	14 (100.0%)	0 (0.0%)	
BMI Category			
Overweight	16 (64.0%)	9 (36.0%)	<0.001 *
Normal weight	40 (93.0%)	3 (7.0%)	
Obese	8 (42.1%)	11 (57.9%)	
Smoke Status			
No	39 (90.7%)	4 (9.3%)	<0.001 *
Current Smoker	25 (56.8%)	19 (43.2%)	
Alcohol			
No	38 (80.9%)	9 (19.1%)	0.095 **
Yes	26 (65.0%)	14 (35.0%)	
HT			
No	27 (73.0%)	10 (27.0%)	0.914 **
Yes	37 (74.0%)	13 (26.0%)	
DM			
No	44 (80.0%)	11 (20.0%)	0.074 **
Yes	20 (62.5%)	12 (37.5%)	
CAD			
No	41 (74.5%)	14 (25.5%)	0.785 **
Yes	23 (71.9%)	9 (28.1%)	

* Fisher’s exact test ** χ^2^ tests. BMI: body mass index (kg/m^2^); HT: hypertension; DM: diabetes mellitus; CAD: coronary artery disease.

**Table 2:** Comparison of preoperative laboratory parameters according to bladder recurrence status.

	Bladder Recurrence		
	No (Mean ± SD)	Yes (Mean ± SD)	*p*-Value
Age	67.9 ± 8.7	65.4 ± 5.0	0.198
Preop Hb	12.1 ± 2.2	11.5 ± 1.8	0.257
Preop WBC	8.81 ± 3.42	7.58 ± 1.52	0.101
Preop PLT	278.2 ± 114.2	291.9 ± 97.1	0.610
Preop Creatinine	1.66 ± 1.53	2.06 ± 1.98	0.324
De Ritis	1.27 ± 0.63	2.09 ± 0.84	<0.001

Independent Sample *t*-test. Hb: hemoglobin, WBC: white blood cell count, PLT: platelet count, De Ritis: AST/ALT.

**Table 3:** Perioperative and pathological features by bladder recurrence status.

	Bladder Recurrence		
	No (n, %)	Yes (n, %)	*p*-Value
UTUC Stage			
Ta	11 (68.8%)	5 (31.3%)	0.335 *
T1	15 (75.0%)	5 (25.0%)	
T2	16 (88.9%)	2 (11.1%)	
T3	21 (65.6%)	11 (34.4%)	
UTUC Grade			
Low	15 (75.0%)	5 (25.0%)	0.784 **
High	46 (71.9%)	18 (28.1%)	
LVI			
No	45 (84.9%)	8 (15.1%)	0.003 **
Yes	19 (55.9%)	15 (44.1%)	
LNI			
No	45 (72.6%)	17 (27.4%)	0.820 **
Yes	18 (75.0%)	6 (25.0%)	
Surgical Margin			
Negative	58 (80.6%)	14 (19.4%)	0.001 **
Positive	6 (40.0%)	9 (60.0%)	
Synchronicity BCa			
No	46 (88.5%)	6 (11.5%)	<0.001 **
Yes	18 (51.4%)	17 (48.6%)	
Synchronicity BCa Number			
Solitary	15 (75.0%)	5 (25.0%)	0.002 *
Multiple	3 (20.0%)	12 (80.0%)	
Synchronicity BCa Stage			
Ta	6 (54.5%)	5 (45.5%)	0.325 *
T1	10 (45.5%)	12 (54.5%)	
T2	2 (100.0%)	0 (0.0%)	
ICM			
No	41 (67.2%)	20 (32.8%)	0.020 *
BCG	21 (95.5%)	1 (4.5%)	
MTC	2 (50.0%)	2 (50.0%)	
RNU Technique			
Open	54 (76.1%)	17 (23.9%)	0.267 **
Laparoscopic	10 (62.5%)	6 (37.5%)	
Bladder Cuff Resection Method			
Open	22 (75.9%)	7 (24.1%)	0.731 **
Endoscopic	42 (72.4%)	16 (27.6%)	

* Fisher’s exact test ** χ^2^ Tests. UTUC: upper tract urothelial carcinoma, LVI: lymphovascular invasion, LNI: lymph node involvement, BCa: bladder cancer, ICM: Intravesical Medication, BCG: Bacillus Calmette-Guérin, MTC: Mitomycin-C, RNU: radical nephroureterectomy.

**Table 4:** Multivariate logistic regression analysis identifying independent predictors of bladder recurrence.

						95% Confidence Interval	
Predictor	Estimate	SE	Z	*p*	Odds Ratio	Lower	Upper
Intercept	−5.67	1.195	−4.75	<0.001	0.00343	3.30 × 10^−4^	0.0357
Smoke Status:							
Current Smoker—No	1.98	0.759	2.60	0.009	7.22299	1.63	31.9898
De Ritis	1.22	0.420	2.91	0.004	3.39365	1.49	7.7283
Surgical Margin:							
Positive–Negative	1.80	0.816	2.21	0.027	6.06388	1.23	29.9952
Synchronicity BC:							
Yes–No	1.90	0.708	2.68	0.007	6.67960	1.67	26.7724

Note. Estimates represent the log odds of “Bladder Recurrence = Yes” vs. “Bladder Recurrence = No”. Model fit indices: Deviance = 57.6, AIC = 67.6, McFadden R^2^ = 0.427, Tjur R^2^ = 0.469.

**Table 5:** Survival outcomes and Cox regression analyses according to De Ritis ratio.

Group	n	Events	Median (Month)	1-Year	3-Year	5-Year	HR (95% CI)	*p*
Recurrence-Free Survival								
De Ritis Low	60	6	Not reached	84.0%	84.0%	84.0%	Reference	–
De Ritis High	27	17	9 (6–NA)	38.9%	0.0%	0.0%	0.08 (0.03–0.22)	<0.001
Cancer-Specific Survival								
De Ritis Low	60	4	72 (72–NA)	97.6%	97.6%	97.6%	Reference	–
De Ritis High	27	9	36 (28–NA)	73.0%	38.9%	19.5%	0.10 (0.03–0.33)	<0.001
Overall Survival								
De Ritis Low	60	44	30 (18–36)	65.7%	33.8%	21.9%	Reference	–
De Ritis High	27	9	30 (12–NA)	54.4%	27.2%	27.2%	1.28 (0.62–2.64)	0.511

Kaplan–Meier and univariate Cox regression analyses. HR: hazard ratio; CI: confidence interval; NA: not available.

## Data Availability

The data presented in this study are available upon reasonable request from the corresponding author. The data are not publicly available due to institutional privacy policies and ethical restrictions.

## Abbreviations

The following abbreviations are used in this manuscript:

UTUC	Upper Tract Urothelial Carcinoma
RNU	Radical Nephroureterectomy
AST	Aspartate Aminotransferase
ALT	Alanine Aminotransferase
ROC	Receiver Operating Characteristic
AUC	Area Under the Curve
RFS	Recurrence-Free Survival
CSS	Cancer-Specific Survival
OS	Overall Survival
BCa	Bladder Cancer
OR	Odds Ratio
CI	Confidence Interval
HR	Hazard Ratio
LVI	Lymphovascular Invasion
